# Effects of Blood Contamination and the Rostro-Caudal Gradient on the Human Cerebrospinal Fluid Proteome

**DOI:** 10.1371/journal.pone.0090429

**Published:** 2014-03-05

**Authors:** Elise Aasebø, Jill Anette Opsahl, Yngvild Bjørlykke, Kjell-Morten Myhr, Ann Cathrine Kroksveen, Frode S. Berven

**Affiliations:** 1 Proteomics Unit (PROBE), Department of Biomedicine, University of Bergen, Bergen, Norway; 2 The KG Jebsen Centre for MS-research, Department of Clinical Medicine, University of Bergen, Bergen, Norway; 3 Department of Clinical Science, University of Bergen, Bergen, Norway; 4 The Norwegian Multiple Sclerosis Competence Centre, Department of Neurology, Haukeland University Hospital, Bergen, Norway; University of Jaén, Spain

## Abstract

Over the last years there has been an increased focus on the importance of knowing the effect of pre-analytical influence on the proteomes under study, particularly in the field of biomarker discovery. We present three proteomics studies examining the effect of blood contamination and the rostro-caudal gradient (RCG) on the cerebrospinal fluid (CSF) proteome, in addition to plasma/CSF protein ratios. The studies showed that the central nervous system (CNS) derived proteins appeared to be unaffected by the RCG, while the plasma-derived proteins showed an increase in concentration towards the lumbar area. This implies that the concentration of the plasma-derived proteins in CSF will vary depending on the volume of CSF that is collected. In the CSF samples spiked with blood, 262 of 814 quantified proteins showed an abundance increase of more than 1.5 fold, while 403 proteins had a fold change of less than 1.2 and appeared to be unaffected by blood contamination. Proteins with a high plasma/CSF ratio appeared to give the largest effect on the CSF proteome upon blood contamination. The results give important background information on how factors like blood contamination, RCG and blood-CNS-barrier influences the CSF proteome. This information is particularly important in the field of biomarker discovery, but also for routine clinical measurements. The data from the blood contamination and RCG discovery studies have been deposited to the ProteomeXchange with identifier PXD000401.

## Introduction

Protein quantification in human body fluids is of high interest in clinical proteomics, and pre-analytical factors may influence the quantitative analysis of the proteome. Knowledge about which and how proteins are affected is important for sample selection and enables exclusion of samples with inadequate quality. Cerebrospinal fluid (CSF), an ultra-filtrate of plasma produced in the choroid plexi, is the most proximal fluid to the central nervous system (CNS) and an important material in the search for biomarkers in neurological diseases. The effect of the pre-analytical influence on the human CSF proteome has previously been studied using proteomics [1]-[4].

Blood contamination is one of the most important pre-analytical factors in CSF analysis. CSF becomes blood contaminated in up to 20% of all lumbar punctures (LP) [5] and due to the high protein concentration in blood, only a small fraction of blood leakage into the CSF will affect the protein concentration, particularly the concentration of blood-derived proteins [5], [6]. Peroxiredoxin, catalase, carbonic anhydrase-I and hemoglobin were found to be blood-contamination specific proteins by You *et al.* [6]. They also suggested that proteases present in blood could cause degradation of CSF proteins in blood contaminated samples. Furthermore, blood proteins have been reported to cause suppression of ion signals in matrix-assisted laser desorption/ionization (MALDI) peptidome analysis of CSF, but this can be reduced by centrifugation of CSF before initial freezing [3], [7]. The general guidelines for selecting high quality CSF recommends to eliminate samples with >500 erythrocytes/μL [4].

As the CSF flows uni-directionally along the spinal cord down to the lumbar subarachnoid space [8], passive diffusion creates a protein concentration gradient from the ventricles to the lumbar area, referred to as the rostro-caudal gradient (RCG). It has been reported that blood-derived proteins such as serum albumin increase down the gradient with up to 2.5 fold change [9], [10] and that proteins that origin from the brain such as NSE, S-100B and tau protein decrease linearly in concentration downwards the RCG [11]. Proteins released from the leptomeninges into the subarachnoid space (prostaglandin-H2 D-isomerase, cystatin-C), have been reported to increase in concentration along the RCG, due to higher local concentration in the surrounding leptomeninges than in the CSF. The concept of a decrease in brain proteins was very recently analyzed by Brandner *et al.* [12] where they analyzed concomitant ventricular and lumbar CSF without confirming the trend described by Reiber [9]. Because of the RCG effect, the protein concentration is higher in the lumbar area than in the ventricular area and due to this it has been recommended to collect a standard volume of CSF during LP [9], [13], [14]. The RCG effect in human CSF has been presented in several studies [9], [11], [12], [15]-[19], but in none using a large-scale proteomics study as presented in this paper.

In the present study, we have examined the effect of blood contamination on the CSF proteome by monitoring the abundance of more than 800 CSF proteins at two different blood spike-in levels through a sodium dodecyl sulfate polyacrylamide gel electrophoresis (SDS-PAGE) LC-MS approach. We also monitored the effect of centrifugation on blood-spiked CSF samples in a related experiment. In a separate experiment, we examined the RCG effect on the CSF proteome using iTRAQ (isobaric tags for relative and absolute quantitation) shotgun proteomics. Multiple reaction monitoring (MRM) MS in combination with stable isotope dilution (SID) was used to monitor 48 proteins derived from blood or CNS (leptomeninges and brain). For this experiment we analyzed CSF from the 2^nd^ to the 45^th^ mL of CSF from one patient with progressive supranuclear palsy (PSP) undergoing LP. In the last experiment, the plasma/CSF ratio was calculated for 152 proteins in five individuals using dimethyl shotgun proteomics. The presented work will be particularly important for quantitative protein measurements in CSF and provides important results for the understanding of the RCG and blood/CSF protein relation.

## Materials and Methods

### Sample collection and handling

CSF was collected from patients undergoing diagnostic LP at the Department of Neurology, Haukeland University Hospital, Bergen, Norway. CSF used in analysis of the CSF/plasma protein ratio was collected from patients undergoing spinal anesthesia at the Department of Orthopedic surgery, Haukeland University Hospital, Norway. The CSF was collected and treated after the guidelines presented in the consensus protocol if not stated otherwise [4]. The plasma samples were collected in K_2_ EDTA tubes and centrifuged at 4°C at 1100×*g* for 10 min. Plasma and CSF was collected at the same time. All CSF and plasma samples went through one freeze/thaw cycle. In the blood contamination study we used fresh blood donated by a healthy volunteer.

### Ethics statement

The study was approved by The Regional Committee for Medical and Health Research Ethics (REC), Western Norway, and written informed consent was obtained from all patients and controls.

### Experimental design

**Figure 1:**
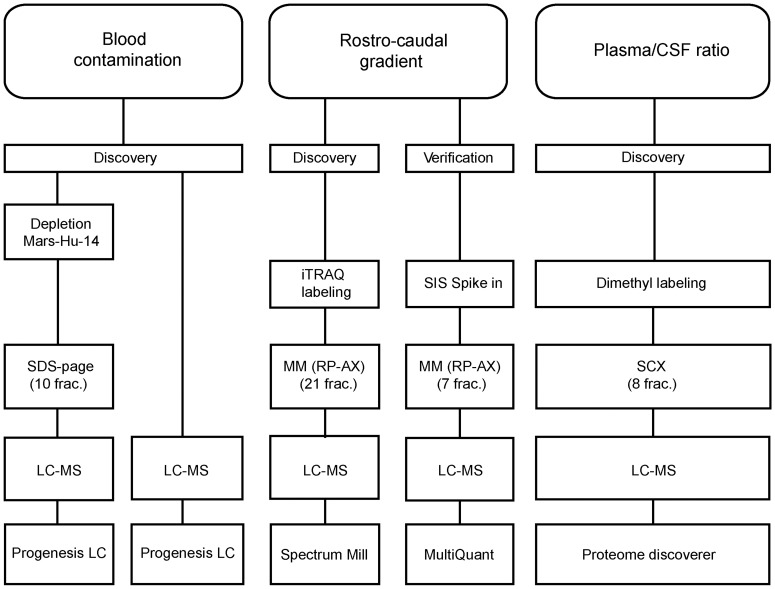
Overview of the conducted studies. Blood contamination: in experiment A protein depleted CSF was separated by SDS-PAGE, in experiment B crude CSF was in-solution digested. Progenesis LC-MS was used for data analysis. In the rostro-caudal gradient study, we used iTRAQ-labeling with mixed mode reversed phase-anion chromatography (MM (RP-AX)) fractionation. The Spectrum Mill software was used for data analysis. For verification we used stable isotope dilution (SID) multiple reaction monitoring (MRM) to monitor 70 peptides. MM (RP-AX) chromatography was used for fractionation and the MultiQuant software was used for data analysis. In the plasma/CSF ratio study equal amount of corresponding CSF and plasma from five patients were compared using dimethyl labeling. Samples were fractionated using strong cation exchange (SCX) chromatography. Proteome discoverer was used for data analysis. For all discovery experiments the samples were analyzed on an Orbitrap Velos Pro and for SID-MRM the samples were analyzed on a Q-trap 5500. SIS = Stable Isotope Standards

The pre-analytical factors we have studied are: Blood contamination of CSF, the RCG of CSF proteins, and plasma/CSF protein ratio (Figure 1).

In the iTRAQ shotgun experiment (Figure 3), 577 proteins were identified (data not shown) of which 279 proteins were quantified with at least two peptides in all experiments (Table S4). Table 1 shows the criteria for distinguishing the effect of the RCG, using the same rational as for the blood contamination study. In addition, we used the R-squared value criteria for selection based on the rational that proteins truly affected by the RCG would show certain linearity in concentration change along the gradient. An R-squared value of >0.7 was considered to reflect sufficient linearity, and in combination with a fold change above 1.5, such proteins were considered to have a measurable quantitative change related to the RCG. By using these criteria we found that 60 proteins were affected by the RCG. For 120 proteins it was difficult to determine whether or not they were affected and these proteins were therefore regarded as uncertain. For the remaining 99 proteins we could not measure any effect of the RCG and they were termed unaffected. Plasma proteins were highly represented among the proteins which increased in concentration towards the lumbar area. Between RCG point 1 and 7 the plasma proteins albumin and haptoglobin had a fold change increase of 1.58 and 1.79. Hemoglobin beta had a fold change of 12.68 between RCG point 1 and 7, but only 1.19 between RCG point 2 and 7. The high abundance of hemoglobin beta in RCG point 1 reflects the high number of erythrocytes (37 000/μL) in the 0-1^st^ mL sample, which was used for routine analysis and thus not included in our dataset. Among the proteins unaffected by the RCG were mainly proteins expected to predominantly arise from the CNS, like cystatin-C and contactin-1, whereas in the uncertain category we found a mixture of both blood- and CNS-derived proteins.

**Table 1:** Categorization of proteins in the rostro-caudal gradient study.

Fold change	R-square	Definition	# Proteins
≥ 1.5	> 0.7	Affected	60
< 1.5	> 0.7	Uncertain	41
> 1.2	0.3-0.7	Uncertain	79
< 1.2	0.3-0.7	Unaffected	44
	< 0.3	Unaffected	55

Fold change and R-squared limits for defining proteins quantified in the rostro-caudal gradient (RCG) iTRAQ experiment as affected, uncertain or unaffected by the gradient. The fold change and R-squared limit is calculated between the 1-2^nd^ and 44-45^th^ mL of CSF, referred to as RCG point 1 and 7.

### Chemicals

Urea, dithiothreitol (DTT), iodoacetamide (IAA), N-octyl-β-D-glucopyranoside (NOG) and calcium chloride were purchased from Sigma-Aldrich (St. Louis, MS, USA). Trypsin Porcine was purchased from Promega. Tris (hydroximethyl) amonimethane was from Merck KGaA (Darmstadt, Germany). Water, acetonitrile (ACN) and formic acid (FA) were MS grade, purchased from Fluka Analytical, Sigma-Aldrich. The iTRAQ kit was purchased from AB SCIEX (Framingham, MA, USA). 4-12% Bis-Tris gel (NuPAGE®), MES SDS Running Buffer, lithium dodecyl sulfate (LDS) sample buffer, and SeeBlue Plus2 Pre-Stained Standard were from Invitrogen, Life Technologies (Carlsbad, CA, USA). Stable Isotope Standards (SISs) were in crude quality unless stated otherwise, and were purchased from Thermo Fisher Scientific (Ulm, Germany) and JPT Technologies (Berlin, Germany). Their C-terminal lysine or arginine was labeled with ^13^C and ^15^N. Formaldehyde-d_2_ (CD_2_O) (98% D, 20 wt %), ^13^CD_2_O (99% ^13^C, 98% D, 20 wt %), and sodium cyanoborodeuteride (96% D) were from Isotec (Miamisburg, OH, USA).

### Analysis of total protein concentration

Total protein concentrations for all samples were measured using Qubit™ Fluorometer (Life Technologies).

### The blood contamination study

**Figure 2:**
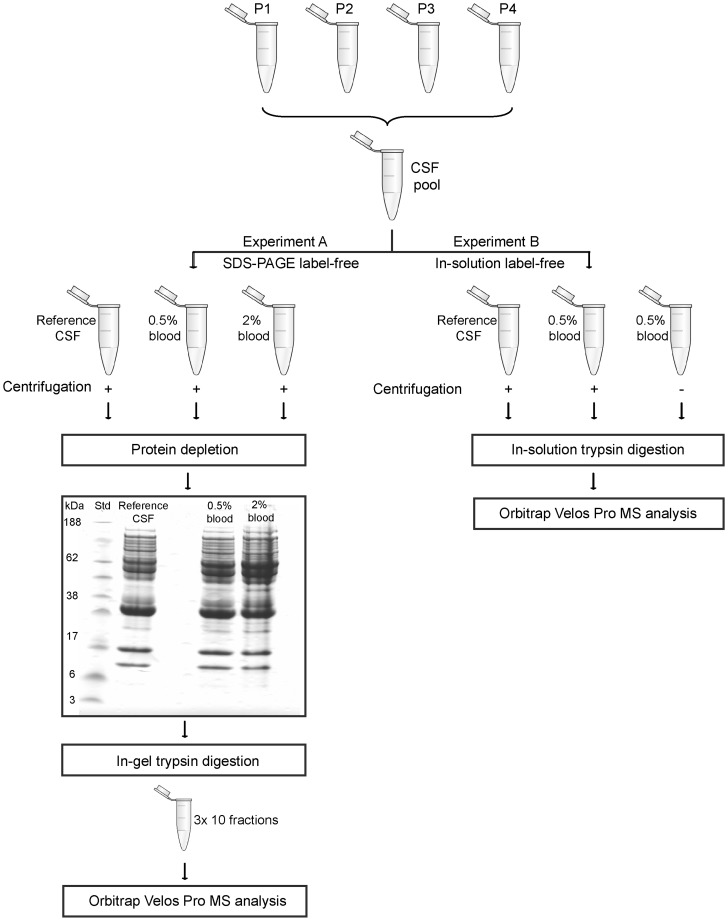
Flow chart of the blood contamination studies. Cerebrospinal fluid (CSF) from four patients (P1-P4) were pooled and divided into aliquots which were spiked with different amounts of blood. In experiment A, a reference sample was compared to CSF spiked to achieve 0.5% and 2% blood. The CSF samples were centrifuged, protein depleted, and separated by SDS-PAGE into ten fractions per lane. In experiment B, we examined the effect of centrifugation. A reference sample was compared to two CSF samples spiked to achieve 0.5% blood, where one of the blood contaminated samples was not centrifuged. The samples were trypsin digested in-solution. All samples were analyzed on an OrbiTrap Velos Pro.

CSF samples with <500 erythrocyte/μL were chosen for the blood contamination experiments. Hemoglobin beta was not detected in any of the CSF samples using SID-MRM (data not shown). One mL CSF from four patients were pooled and divided into four aliquots. One aliquot (1 mL) was kept as reference CSF without added blood, one was spiked with 20 µL blood/mL CSF (2%) and two were spiked with 5 µL blood/mL CSF (0.5%). The sample spiked with 2% blood and one of the samples spiked with 0.5% blood were centrifuged at 4°C at 400×*g* for 10 min. The samples were analyzed in experiment A (SDS-PAGE label-free) and B (in-solution label-free) with experimental setup as shown in Figure 2.

#### Experimental workflow experiment A

CSF was protein depleted according to the manufacturer's protocol using the Multiple Affinity Removal System (MARS-HU-14) 4.6×100 mm LC column (Agilent Technologies, CA, USA) coupled to Dionex Ultimate 3000 LC system (Thermo Scientific, Bremen, Germany). The total protein amount ranged from approximately 500-1400 µg prior to depletion due to different amount of added blood. When depleting five identical aliquots from the same sample, the %CV of the measured protein concentration over all samples was 6.8%. The protein depleted CSF was concentrated using 3 kDa ultra-centrifugation filters (Amicon Ultra-4, Millipore, Bedford, MA) which were pre-rinsed with 0.1% NOG for increased protein recovery, and freeze dried. The pellet was dissolved in 2X LDS sample buffer (NuPAGE, Invitrogen), reduced with 10 mM DTT and alkylated with a final concentration of 20 mM IAA for 20 min in darkness before the samples were loaded on 4-12% gradient Bis-Tris gels (NuPAGE Invitrogen, Life Technologies). Each lane was cut into ten fractions which were washed, trypsin digested, and peptide eluted essentially as described elsewhere [20]. All fractions resulting from the extraction of peptides were combined in the elution plate and freeze dried. The dry peptide mixtures were re-suspended in 0.1% FA and loaded onto a pre-column (Dionex, Acclaim PepMap Nano Trap column, C18, 75 µm i.d. ×2 cm, 3 µm) followed by separation on the analytical column (Dionex, Acclaim PepMap100 LC nano column, 75 µm×15 cm, C18, 2 µm) using a Dionex Ultimate 3000 rapid separation (RS) LC nano system (Thermo Scientific) coupled online to an Orbitrap Velos Pro mass spectrometer (Thermo Scientific, Bremen, Germany). A 90 min LC-method with a gradient composition of mobile phase A (0.1% FA/2% ACN) and mobile phase B (0.1% FA/90% ACN) was used. The gradient was as follows: 5% B from 0-6 min, increasing to 8% B from 6-6.5 min, 8-38% from 6.5-67 min, then 38-90% B from 67-70 min. Hold at 90% B for 5 min, and ramp the gradient from 90-5% B from 75-78 min. Hold at 5% B for the last 12 min. The peptides were eluted at a flow rate 280 nL/min. The mass spectrometer was operated in the data-dependent-acquisition mode to automatically switch between full scan MS and MS/MS acquisition. Instrument control was through Tune 2.7 and Xcalibur 2.2.

Survey full scan MS spectra (from 300 to 2000 *m/z*) were acquired for 80 min in the Orbitrap with a resolution R = 60 000 at 400 *m/z* (after accumulation to a target value of 1e^6^ in the linear ion trap with maximum allowed ion accumulation time of 500 ms). The seven most intense eluting peptides above an ion threshold value of 1000 counts, and charge states ≥ +2, were sequentially isolated to a target value of 1e^4^ (maximum accumulation time was 200 ms) and fragmented in the high-pressure linear ion trap by low-energy CID (collision-induced-dissociation) with normalized collision energy of 40% and wideband-activation enabled. The isolation width was maintained at 2 Da, activation q = 0.25, and activation time of 10 ms. The resulting fragment ions were scanned out in the low-pressure ion trap at normal scan rate, and recorded with the secondary electron multipliers. One MS/MS spectrum of a precursor mass was allowed before dynamic exclusion for 20 s. Lock-mass internal calibration was not enabled. The raw files were analyzed and pre-processed using the Progenesis LC-MS software 2.7 (Nonlinear Dynamics, Newcastle, UK). The MS/MS spectra were analyzed using the database *homo sapiens* SwissProt (downloaded April 2012, 40 486 entries) using the open-source graphical user interface SearchGUI (version 1.7.3) [21], with search engines OMSSA (2.1.9) and X!Tandem (Cyclone, (2010, 12.01.1)) [22], [23]. The following search parameters were used; 10 ppm parent ion tolerance, 0.7 Da fragment mass tolerance, carbamidomethylation of Cys as fixed modification, oxidation of Met as variable modification, and two missed cleavages were allowed. The peptides were assembled to proteins using PeptideShaker (version 0.14.7) (http://peptide-shaker.googlecode.com), and filtered at the stringent threshold of 1% false discovery rate (FDR). The FDR was estimated using the reversed version of the database [24]. Identification results were further processed in the Progenesis software to generate a protein report. The protein report was generated with no protein grouping and quantification was based on non-conflicting features, which means that only unique peptides contributed to quantification.

#### Experimental workflow experiment B

CSF was in-solution digested according to the protocol in Document S1 and analyzed and quantified as described for Experiment A.

### The rostro-caudal gradient experiment

To detect a possible RCG of proteins we sampled the seven following points of the RCG from a PSP patient: 1-2^nd^, 10-11^th^, 16-17^th^, 24-25^th^, 31-32^nd^, 38-39^th^ and 44-45^th^ mL CSF, referred to as RCG point 1-7, respectively. The CSF was centrifuged at 2000×*g* for 10 min, and stored at -80 °C. The protein concentration varied from 0.675-0.414 µg/μL between RCG point 1 and 7 (lumbar to ventricular area). The 0-1^st^ mL was blood contaminated (erythrocytes 37 000/μL); this one mL was used for routine analysis and was not included in this study. The workflow for the RCG experiment is shown in Figure 1.

#### iTRAQ discovery

**Figure 3:**
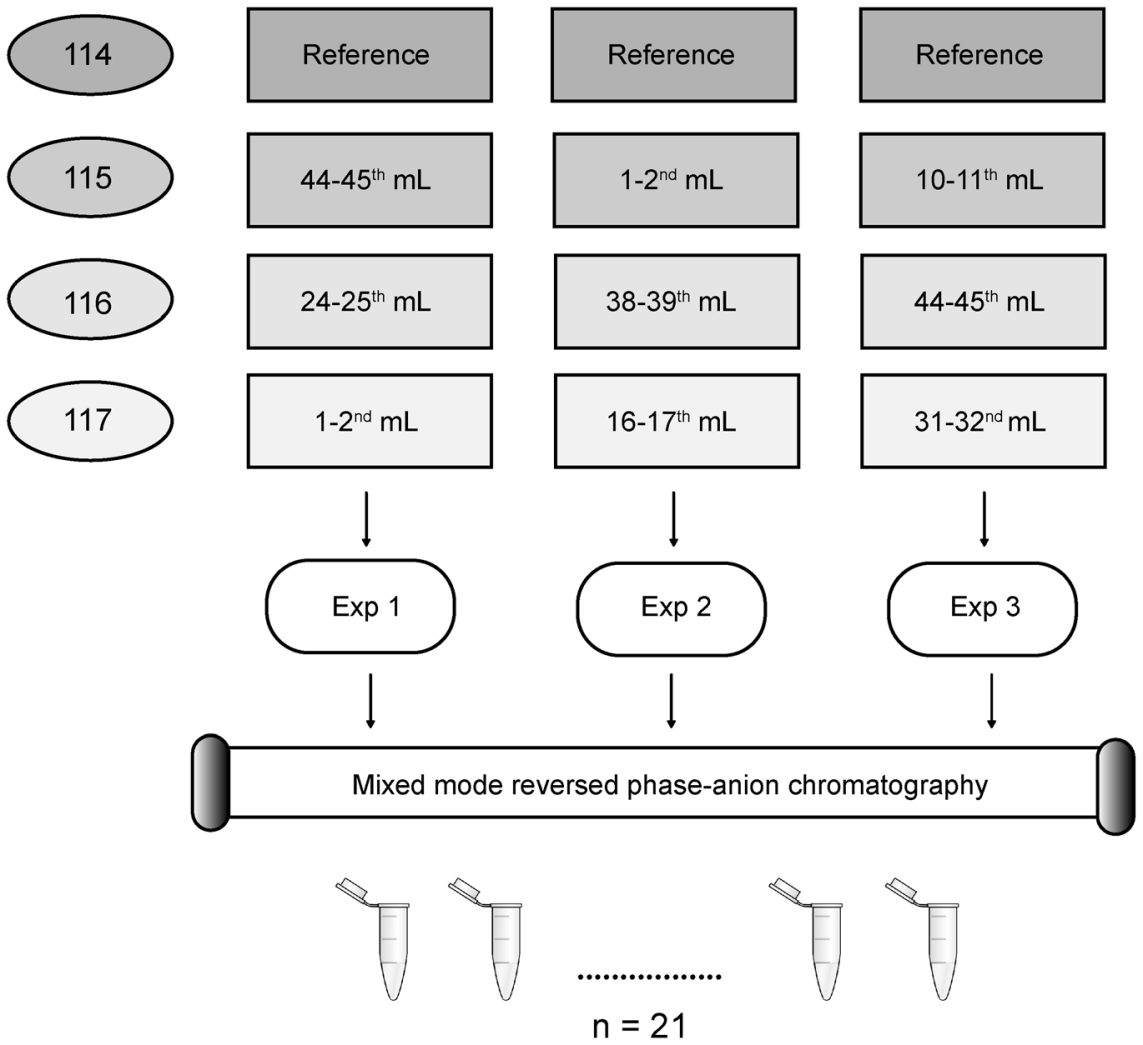
Flow chart of the rostro-caudal gradient study. In the rostro-caudal gradient (RCG) study, we examined the seven following points of the RCG from a PSP patient: 1-2^nd^, 10-11^th^, 16-17^th^, 24-25^th^, 31-32^nd^, 38-39^th^ and 44-45^th^ mL CSF, referred to as RCG point 1-7, respectively. Twelve samples were digested and iTRAQ labeled (114-117). A reference, (labeled with iTRAQ reagent 114) containing the same amount of each RCG point, was included in each experiment. The RCG points 1 and 7 were included twice. After digestion and iTRAQ-labeling, samples were combined as follows: Exp. 1 (reference, 44-45^th^ mL, 24-25^th^ mL and 1-2^nd^ mL), Exp. 2 (reference, 1-2^nd^ mL, 38-39^th^ mL and 16-17^th^ mL), and Exp. 3 (reference, 10-11^th^ mL, 44-45^th^ mL and the 31-32^nd^ mL). The three experiments were fractionated by mixed mode reversed phase-anion chromatography (MM (RP-AX)) and analyzed on an Orbitrap Velos Pro. The protein abundances were averaged for each protein in the duplicate samples.

In the iTRAQ discovery experiment equal volumes representing the seven RCG points were processed as shown in the experimental setup in Figure 3. The three reference samples were identical and contained equal volumes of the seven RCG points. The first and seventh RCG points were included twice in the experimental design. The twelve samples (Figure 3) were dried, re-suspended, and trypsin digested using the iTRAQ® Reagent 4plex Applications Kit according to the vendor's protocol with one exception: 1/3 of the iTRAQ Dissolution Buffer and 1/3 of the iTRAQ® reagents were used for each experiment (n = 3).

The combined and labeled tryptic peptides were dried and fractionated by mixed mode reversed phase-anion exchange (MM (RP-AX)) chromatography on a 1260 Infinity LC system (Agilent, Santa Clara, CA, USA) using a Promix MP column (1.0 mm×250 mm, 5µm, 300 Å) (SIELC Technologies, IL, USA). The duration of the gradient was 70 min, with 50 µL/min flow, using the following buffers: Buffer A (20 mM ammonium formate/3% ACN, pH 6.5) and Buffer B (2 mM ammonium formate/80% ACN, pH 3.0). The dry peptide mixture was re-suspended in Buffer A and fractionated into 21 fractions with the following gradient: Hold at 15% B from 0-10 min. From 10-45 min 15-60% B, from 45-55 min 60-100% B, hold at 100% B from 55-60 min, and ramp from 100-15% B from 60-65 min, then hold at 15% B from 65-70 min. The fractions were freeze-dried and re-suspended in 0.1% FA before injection on the Orbitrap system. MM (RP-AX) fractions 1-4 were excluded, as they were found to contain excess iTRAQ reagents, and the two latest fractions were combined before injection.

Approximately 0.5 µg of each MM (RP-AX) fraction was loaded on an Ultimate 3000 RSLC nano system online connected to an Orbitrap Velos Pro using the same settings and columns as described for the blood contamination study. The seven most intense eluting peptides above an ion threshold of 1000 counts and charge states ≥ +2, were sequentially isolated in a back-to-back analysis of the same precursors using two different fragmentation techniques, CID and HCD (higher-energy collision dissociation). The CID fragmentation was performed as described for the blood contamination study except collision energy of 35% compared to 40%. For the HCD fragmentation, the ions were isolated in the high-pressure linear ion trap to a target value of 5e^5^ at a maximum allowed accumulation time of 1000 ms and isolation width at 3 Da. Fragmentation in the HCD cell was performed with a normalized collision energy of 40%, and activation time of 0.1 ms. Fragments were detected in the Orbitrap at a resolution of 7500 with first mass fixed at *m/z* 160. Two MS/MS spectra of a precursor mass were allowed before dynamic exclusion for 10 s, and lock-mass internal calibration was not enabled.

The generated raw files were extracted, searched and validated using the Spectrum Mill software package v4.0 beta (Agilent Technologies, Santa Clara, CA, USA). The data extraction settings were as follows: Fixed modifications were methyl methanethiosulfonate (MMTS) of Cys and iTRAQ (N-term, Lys), variable modifications were oxidation of Met, fragment mass tolerance MH+ interval 300-4000, scan time range 0-300 min, and sequence tag length >0. Merge CID and HCD MS/MS, and find precursor ^12^C with maximum charge 7. The resulting data were searched against the *homo sapiens* SwissProt database (downloaded August 2010, containing 20 286 entries) with following search parameters: Precursor mass tolerance 10 ppm and fragment mass tolerance 0.7 Da, maximum two missed cleavages allowed, and MMTS (Cys) and iTRAQ (N-term, Lys) as fixed modifications. Reverse database scores were calculated. The default fixed auto validation settings were applied for both proteins and peptides, including calculate FDR using reverse hits. In protein mode, the threshold score was >20, proteins were accepted at precursor charge +2 if score >6, Scored Percent Intensity >60%, delta rank1-rank2 >2, and at charge +3 if >8, >70%, >2. The FDR was <1%. The first and seventh RCG points were included twice in the experimental design and the average protein abundances were reported. In Spectrum Mill, a ratio was calculated for every protein by dividing the protein abundance in an individual RCG sample by the abundance in the reference sample. Using these ratios, we calculated the R-squared value and the fold change values for every protein between the different RCG points.

#### SID-MRM verification in the RCG study

To verify the findings from the RCG iTRAQ discovery, MRM assays were built. Equal volumes of CSF from the seven RCG points were reduced, alkylated and trypsin digested according to the protocol available as Document S1. The SIS mixture was added to the digest with concentrations corresponding to the endogenous peptides and desalted with the Oasis HLB *µ*Elution 96 well plate (Waters, Milford, MA) as described in [25]. The spiked CSF digests were divided into seven fractions using MM (RP-AX) with the same LC gradient as for the iTRAQ-labeled RCG samples.

The samples were re-suspended in 3% ACN/5% FA, and equal volumes of each sample (ranging from 0.6-1 µg) were analyzed on a triple quadrupole tandem mass spectrometer (Q-TRAP 5500 (AB SCIEX, MA, USA)) online coupled to a Dionex Ultimate 3000 RSLC nano system. The spiked CSF digest was loaded onto the pre-column and analyzed using the same MS settings and LC gradient as described elsewhere [25]. The final MRM assay contained 70 peptides corresponding to 48 proteins, and at least three transitions were monitored for each peptide. The most intense transition free of interference was used for quantification. The complete list of proteins, peptides, Q1 and Q3 values and parameter settings is available in Table S1. The MRM data were analyzed in MultiQuant 2.0.2 (AB SCIEX) using the same settings as described previously [25]. To detect possible interference, a fractionated CSF test sample was run three times and analyzed using the open-source software AuDIT [26]. The % coefficient of variance (CV) of the pipeline system was found to be <20 by analyzing an unfractionated CSF sample as quality control between every seventh sample (data not shown). The ratio between the SIS and endogenous peptide was calculated as previously described [25]. This ratio was used to calculate the fold change and the R-squared value for all the included proteins across the seven different points in the RCG.

### The plasma/CSF protein ratio experiment

**Figure 4:**
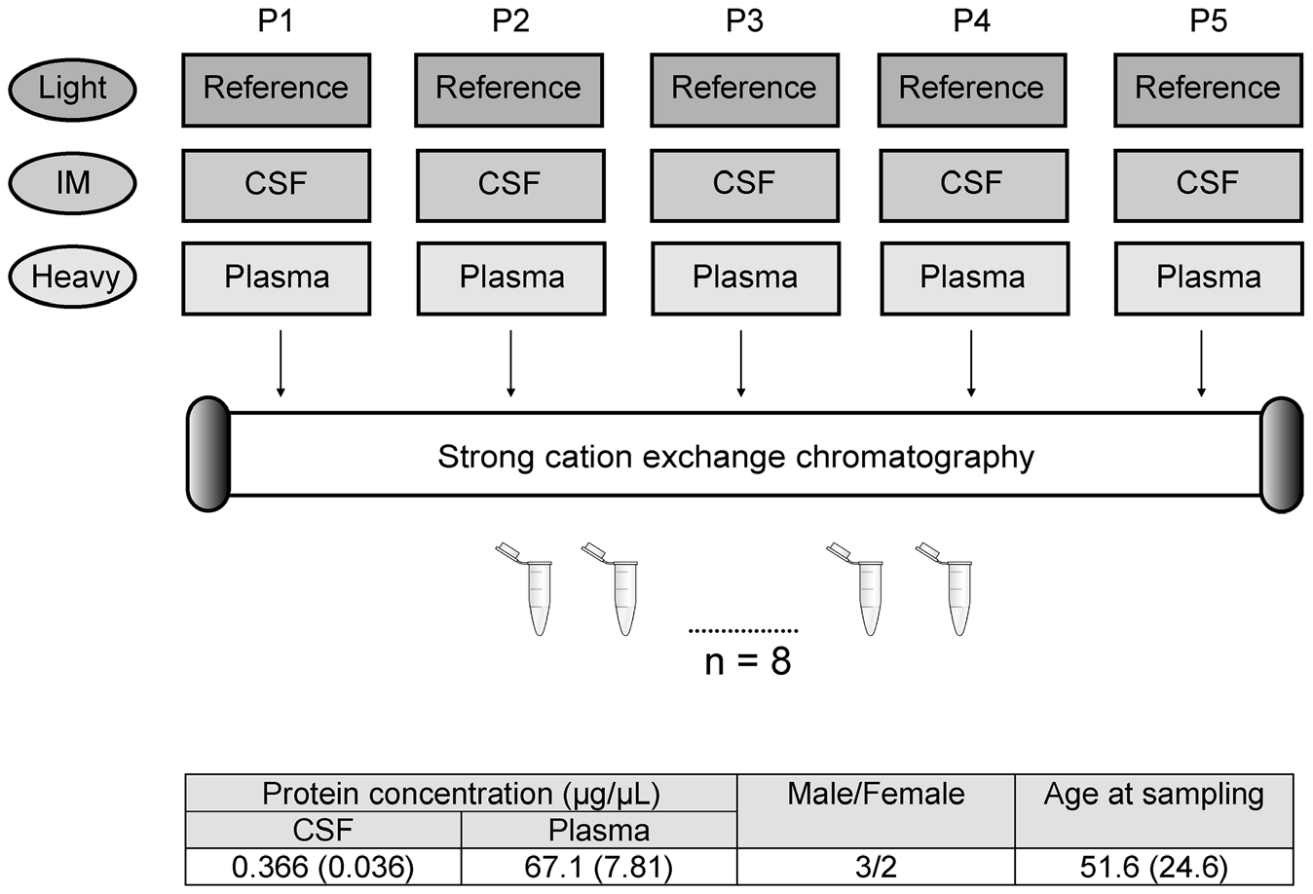
Flow chart of the plasma/CSF study. We compared the cerebrospinal fluid (CSF) and plasma protein ratio of five patients (P1-P5) using dimethyl labeling. The reference sample was a mix of equal total amount of CSF and plasma, and was labeled by light reagents. The five CSF samples were labeled by intermediate (IM) reagents, and the plasma samples were labeled by the heavy reagents. The light, IM and heavy labeled samples were combined and fractionated into eight fractions by strong cation exchange chromatography and analyzed on an Orbitrap Velos Pro. The average (and standard deviation) protein concentration of CSF and plasma, age at sampling and ratio male/female of the five patients are presented in the figure.

To investigate the plasma/CSF ratio for a larger number of proteins we collected corresponding plasma and CSF from five patients undergoing surgery using spinal anesthesia. These patients had no neurological history and did not have any inflammatory diseases. None of the CSF samples had visible amounts of blood, and hemoglobin beta was not detected by SID-MRM. The abundance of proteins present in both plasma and CSF were quantified using dimethyl labeling. A reference mix was prepared of total 10 µg protein from both plasma and CSF from each of the five patients. The individual samples were digested and labeled as shown in Figure 4. The digestion and labeling protocol is described elsewhere [27]. The samples were analyzed using the Orbitrap Velos Pro as explained for the blood contamination study, with some exceptions. A 50 cm analytical column (Acclaim PepMap 100, 50 cm×75 µm i.d. nanoViper column, packed with 3 mm C18 beads) was used, and the gradient composition was 5-8% B over 1 min, then 8–35% B over 134 min followed by 35–90% B over 15 min. Elution of very hydrophobic peptides and conditioning of the column were performed during 15 min isocratic elution with 90% B and 20 min isocratic elution with 5% B, respectively. The raw files were analyzed patient vice in Proteome discoverer 1.3 (Thermo, Bremen, Germany) using the following work flow: From spectrum files to event detector and precursor ions quantifier for quantification and spectrum files to spectrum selector, Mascot and Perculator for identification by searching against the reviewed SwissProt *homo sapiens* (downloaded January 2013, 20 226 entries). The following settings were used: Carbamidomethylation of Cys as fixed modification, oxidation of Met as variable. 10 ppm for precursor and 0.7 Da for fragment tolerance, two missed cleavages were allowed, and FDR was set to 1%. Only unique peptides were used for quantification and only proteins with two or more peptides in two or more patients were included. The abundance of every protein was calculated in every sample by Proteome Discoverer, and a ratio was calculated by dividing the abundance in the plasma sample by the abundance in the corresponding CSF sample. This ratio was used to calculate an average ratio and a %CV for every protein across the five individuals.

## Results

### Effect of blood contamination

To investigate how blood contamination affects the CSF proteome, a reference CSF sample was compared to CSF spiked to achieve 0.5% and 2% blood (experiment A, Figure 2). A total of 1869 proteins were identified and 814 proteins were quantified with two unique peptides or more (Table S2). Even though the samples were protein depleted, small levels of albumin, fibrinogen, apolipoprotein AI and AII, complement 3 and transthyretin remained in the sample. Their quantitative data were omitted from the results.

The quantitative information for the 814 proteins for the two comparisons (1) reference versus 0.5% blood and (2) reference versus 2% blood, are given in Table S2. The 262 proteins with an abundance increase of 1.5 fold in either of the two spike-in levels were defined as affected by the blood contamination, while 403 proteins had a fold change of less than 1.2 in both experiments and were defined as not affected. The 149 proteins with observed fold changes between 1.2 and 1.5 were defined as uncertain with respect to being affected by the blood contamination. A protein abundance change of less than 20% is difficult to detect with the semi-quantitative methods applied as the technical variance is considered to be at least 20%. Hence, all proteins with less than 20% abundance change in our dataset were termed “unaffected”. The proteins with an abundance change of more than 50% are considered highly likely to be affected by the blood contamination, even though false positives cannot be ruled out. These proteins were termed “affected”. When there is an observed fold change of 20-50%, it is difficult to determine whether a protein is affected by the added blood or if the observed change is due to technical inaccuracies. Hence, this category was termed “uncertain”. Naturally, there were many known blood-proteins among the affected proteins. The most affected proteins appeared to be those associated to, or found in red blood cells, platelets or other larger components in blood. Examples of such proteins are purine nucleoside phosphorylase (123 increase in fold change), platelet factor 4 (57 increase in fold change) and apolipoprotein C-II (51 increase in fold change). For proteins secreted by the liver, a typical four fold increase was observed in the CSF sample spiked to achieve 2% blood (complement factor H (3.98), plasma kallikrein (4.43) and serum amyloid A-4 protein (4.61)). In the unaffected group we found as expected mainly CNS derived proteins.

To examine how blood contaminated CSF is affected by centrifugation, we compared two CSF samples spiked to achieve 0.5% blood where only one was centrifuged (experiment B, Figure 2). We found that 54 out of 129 quantified proteins increased more than 1.5 fold in the not-centrifuged sample and these were regarded as affected by centrifugation (Table S3). More than 10 fold increase was observed for 20 proteins and among these were proteins previously suggested as markers for blood contamination like hemoglobin and carbonic anhydrase 1 and 2 [6]. Our results showed that centrifugation before freezing removed most of the amount of these proteins.

### Proteins affected by the rostro-caudal gradient

In this study we used shotgun and targeted proteomics to quantify peptides and proteins in seven RCG points ranging from the 2^nd^ - 45^th^ mL CSF collected by LP from one individual. The first aim was to examine whether the protein origin (plasma or CNS) reflects the protein concentration change along the RCG as previously suggested [11]. Another aim was to explore whether different sampling volumes of CSF could affect the concentration of proteins, which is highly relevant in designing proteomic biomarker studies.

**Figure 5:**
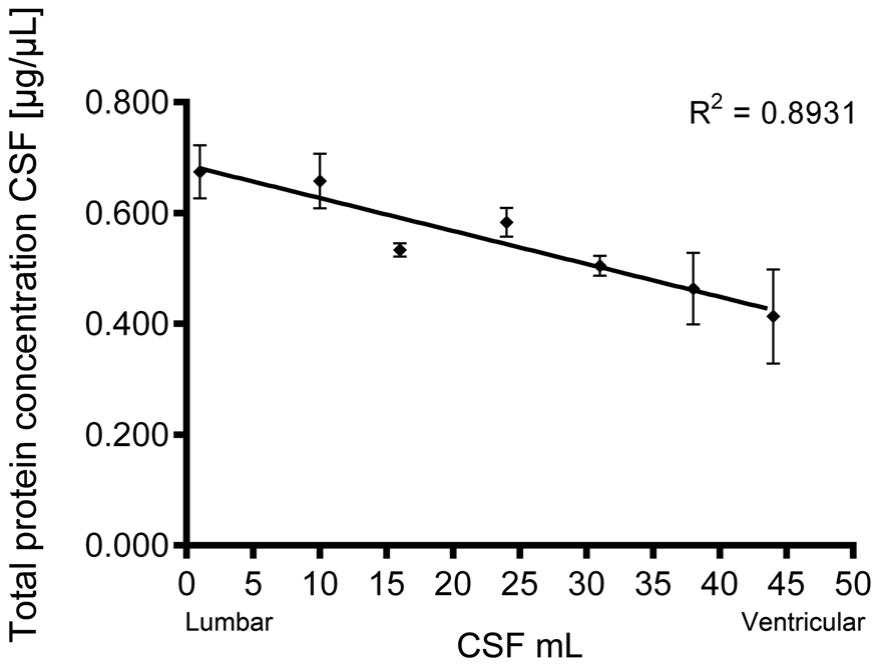
Protein concentration measurement of along the rostro-caudal gradient. Protein concentration of the cerebrospinal fluid (CSF)-derived proteins from the seven points along the rostro-caudal gradient (RCG). The CSF was measured in triplicates with Qubit, and error bars of the standard deviation are included. R squared value 0.8931.

The fold change of the overall protein concentration between RCG point 1 (the 1-2^nd^ mL) and 7 (the 44-45^th^ mL) was 1.63, indicating an approximate 60% total protein concentration decrease from the lumbar towards the ventricular area. These results show a good linearity with an R-squared value of 0.89 (Figure 5) and correlate to previous studies [14]. To verify the change in total protein concentration depending on the volume of CSF collected, the CSF protein concentration between the 1^st^ and 20^th^ mL of the RCG was measured in two new patients (with free CSF circulation). The fold change of the protein measurements were 1.3 and 1.45 (data not shown), verifying the trend observed from the first patient.

A SID-MRM study of 48 proteins (70 peptides) was designed to further confirm the trend observed during the iTRAQ quantification (Table S5). Based on the criteria from Table 1 the proteins quantified in the two related experiments were again categorized according to whether they were affected by the RCG or not, in addition to being classified into CNS-derived (origins from neurons, glial and leptomeninges) and blood-derived based on literature mining (Table 2). The results from the iTRAQ and SID-MRM study showed that plasma-derived proteins increased in concentration downwards in the RCG whereas there was no observable abundance change for the CNS-derived proteins. In both the iTRAQ and SID-MRM study the fold change was found to be typically around 1.6 for the plasma-derived proteins between RCG point 1 and 7 (Table S5). Proteins produced in the leptomeninges such as prostaglandin-H2 D-isomerase, are considered by others to increase down the RCG due to a constant production rate. In our experiment we found that prostaglandin-H2 D-isomerase had a fold change of 1.2 in the MRM and 1.3 in the iTRAQ study between RCG point 1 and 7, and the protein was placed in the uncertain group (Table S5). Proteins with mixed origins such as transthyretin are suggested to increase down the RCG. With a fold change of 1.2 in the iTRAQ experiment and 1 in the MRM experiment we concluded that there was no observable change for this protein.

**Table 2:** Categorization of proteins based on fold change, R-squared values and major expected contributing source of origin.

Acc. No	Protein ID	Category	Expected contributor
P01009	Alpha-1-antitrypsin	Affected	Blood
P02647	Apolipoprotein A-I	Affected	Blood
P08603	Complement factor H	Affected	Blood
P00738	Haptoglobin	Affected	Blood
P02750	Leucine-rich alpha-2-glycoprotein	Affected	Blood
P04004	Vitronectin	Affected	Blood
P02768	Serum albumin	Affected	Blood [9]
P02763	Alpha-1-acid glycoprotein 1	Affected*	Blood
P01011	Alpha-1-antichymotrypsin	Affected*	Blood
P00450	Ceruloplasmin	Affected*	Blood
P01024	Complement C3	Affected*	Blood
Q14624	Inter-alpha-trypsin inhibitor heavy chain H4	Affected*	Blood
P02743	Serum amyloid P-component	Affected*	Blood
P01023	Alpha-2-macroglobulin	Uncertain	Blood
P68871	Hemoglobin subunit beta	Uncertain	Blood
P02787	Serotransferrin	Uncertain	Blood
P69905	Hemoglobin subunit alpha	Uncertain^a^	Blood
P41222	Prostaglandin-H2 D-isomerase	Uncertain	CNS (lept.) [9]
P05090	Apolipoprotein D	Uncertain	Several tissues
P06396	Gelsolin	Uncertain	Several tissues
P05067	Amyloid beta A4 protein	Unaffected	Several tissues
P36222	Chitinase-3-like protein 1	Unaffected	Several tissues
P10909	Clusterin	Unaffected	Several tissues
O43505	N-acetyllactosaminide beta-1,3-N-acetylglucosaminyltransferase	Unaffected	Several tissues
Q8WXD2	Secretogranin-3	Unaffected	Several tissues
O75326	Semaphorin-7A	Unaffected	Several tissues
P02649	Apolipoprotein E	Unaffected	Several tissues
P43121	Cell surface glycoprotein MUC18	Unaffected*	Several tissues
P36955	Pigment epithelium-derived factor	Unaffected*	Blood
P49908	Selenoprotein P	Unaffected*	Blood
P02766	Transthyretin	Unaffected*	Both [9]
P01034	Cystatin-C	Unaffected	CNS (lept.) [9]
O15240	Neurosecretory protein VGF	Unaffected	CNS/PNS
P51693	Amyloid-like protein 1	Unaffected	CNS
Q96GW7	Brevican core protein	Unaffected	CNS
Q12860	Contactin-1	Unaffected	CNS
Q92876	Kallikrein-6	Unaffected	CNS
P13591	Neural cell adhesion molecule 1	Unaffected	CNS
Q99574	Neuroserpin	Unaffected	CNS
Q9UHG2	ProSAAS	Unaffected	CNS
P05060	Secretogranin-1	Unaffected	CNS
P13521	Secretogranin-2	Unaffected	CNS
P10645	Chromogranin-A	Unaffected	CNS [31]
Q9P121	Neurotrimin	Unaffected	CNS [32]
Q99435	Protein kinase C-binding protein NELL2	Unaffected	CNS [33]
Q9P0K1	Disintegrin and metalloproteinase domain-containing protein 22	Unaffected*	CNS
O95502	Neuronal pentraxin receptor	Unaffected*	CNS [34]
P04271	Protein S100-B	Unaffected^a^	CNS (brain) [9]

In order to explore how different proteins were affected by the rostro-caudal gradient, we used the fold change and R-squared values of the proteins quantified with SID-MRM and iTRAQ to categorize the proteins into the three categories: affected by the RCG, unaffected by the RCG and uncertain. The fold change was calculated between the 1-2^nd^ and 44-45^th^ mL of CSF (referred to as RCG point 1 and 7), and the classification was based on the criteria from Table 1.The proteins are also categorized into groups based on the major expected contributing source of origin, with Uniprot as the primary reference unless otherwise stated. See Table S5 for details. The asterisk (*) marks conflicting results: affected + unaffected  =  uncertain; affected + uncertain  =  affected; unaffected + uncertain  =  unaffected. Proteins only quantified in the SID-MRM study is marked with ^a^.

### Plasma/CSF protein ratio

Corresponding plasma and CSF samples from five individuals without neurological symptoms were analyzed by dimethyl-based shotgun proteomics using an Orbitrap Velos Pro. Our goal was to obtain estimates on the plasma/CSF ratios for a portion of the proteins present in these proteomes. This information could be used to further indicate which CSF proteins that appears to be affected by or origins from plasma/blood. It will also give information about these proteins ability to cross the blood-brain barrier (BBB), and indicate if it would be useful to quantify a given biomarker candidate protein in both CSF and plasma in a biomarker verification experiment. Equal total amount of protein (10 µg) from CSF and plasma was par-wise compared for the five patients, and the plasma/CSF ratio for each protein was averaged across the five individuals. From 434 proteins identified across all experiments (data not shown), 152 proteins were quantified with two or more unique peptides in two or more individuals (Table S6). Albumin had an average plasma/CSF ratio of 1.03, ranging from 0.74 to 1.52 (%CV 35.6) (Table S6). These ratios are based on comparison of equal total protein amounts of plasma and CSF proteins. If calculated based on equal volume, the average albumin plasma/CSF ratio was 177∶1. This is in accordance to the expectation that the plasma to CSF protein concentration is approximately 200∶1. The blood proteins showed a varying plasma/CSF ratio with distributions from around 1 all the way to 81. The proteins with the largest plasma/CSF ratio were fibrinogen, apolipoprotein B-100 and Ig mu chain C region, all with ratios between 48 and 81, while hemoglobin alpha had a ratio of about 6. In addition to these proteins, most of the other blood-derived proteins also appeared to have a higher plasma/CSF ratio compared to albumin, indicating that they do not cross the blood-CNS barriers to the same degree as albumin.

### Combining the information from the different experiments

Table S7 combines all the results from the shotgun experiments described in the three previous sections (Table S2, S3, S4 and S6 combined). All the data can easily be combined since only unique peptides have been associated with their respective protein accession numbers. Combining proteomic data in this format is possible when different search engines have been used since there will not be any conflict with peptides shared between several accession numbers leading to protein grouping. The exception from this is the RCG iTRAQ experiment where protein groups have been used. When one or more of the peptides map to more than one accession number, then all these numbers are listed in the table (column: acc.numbers_RCG_exp.). In cases with a group of proteins, the leading protein accession number has been aligned with the corresponding accession numbers from the other experiments. Proteins with unique peptides and peptides that also match other accession numbers, are listed as single entries with an indicator of having shared peptides (column: subgroupNum_RCG_exp). This table gives a simple and complete overview of the proteins we detected in our experiments and whether they are affected by blood spike–in, centrifugation, RCG, their plasma/CSF ratio and also how the relation is between the experiments for each protein.

### Suggested biomarker candidates could be affected by blood contamination

It is known that several neurological disorders lead to impaired BBB which may cause increased influx of plasma proteins into CSF, and in addition, CSF can be blood contaminated during LP. Comparing the list of 201 proteins previously reported to be differentially abundant in CSF of multiple sclerosis patients (reviewed in [28]) with the combined list in Table S7 allowed 79 matches. Of these, we found 35 proteins to potentially be affected by blood contamination, 23 were unaffected and 14 were uncertain. This indicates that perhaps 50% or more of the proteins previously reported as candidate biomarkers for multiple sclerosis are potentially affected by blood contamination, and their origin, at least in part, could be from plasma. One possible reason for the high number of apparently plasma-derived proteins being reported as regulated in multiple sclerosis is that the BBB could be affected in these patients and this again could influence the degree of influx of plasma proteins into their CSF.

## Discussion

If the concentration of a biomarker candidate is influenced by blood contamination, then the quantitative measurements of this protein should be assessed with extra caution. The information from the blood contamination experiments will be highly relevant to determine whether blood contaminated CSF samples are acceptable to be included in a verification study. If your protein of interest is unaffected by blood contamination, then blood contaminated samples could potentially still be used in verification studies of this protein. In relation to this, the results also indicate which proteins in CSF that origin (fully or partially) from plasma, and which proteins that appear to be unaffected by blood contamination and are likely to be CNS-derived. The correct endogenous amounts of the latter category of proteins could likely be measured in CSF even if the samples are blood contaminated. Some of the proteins observed in the unaffected group in Table S2 appeared to decrease in amount after blood spike-in, as 45 proteins had a more than two fold decrease in the samples spiked to achieve 2% blood. This was however not observed for the samples spiked to achieve 0.5% blood. Hence, we hypothesize that the addition of large amounts of blood to the CSF samples and analysis by label-free shotgun proteomics could lead to suppression of lower abundant proteins and that their abundances appear to be lower than they are. One could also envision that the addition of blood would introduce new proteases that degrade certain proteins, as has been suggested previously [2], [6]. Some proteins are synthesized locally in the CNS in addition to arising from plasma, leading to a mixed contribution to the CSF concentration of these proteins. The “uncertain” category in the blood contamination experiment (Table S2) is particularly likely to contain such proteins, as there seems to be some effect of blood contamination for these proteins, but not sufficient to say for sure that there is an effect. Even if a protein mainly arises from plasma, its migration across the BBB might be variable due to inter-individual or disease related variations in the BBB properties. Hence, for most proteins detected in both CSF and plasma, it would be useful to measure the concentration in both fluids. During depletion, proteins not targeted by the column could be removed due to unspecific binding to the column or by binding to target proteins. The blood contamination study included analysis of both depleted (Experiment A) and non-depleted (Experiment B) samples. The information in Table S2 and S3 is therefore complementary, and one can examine if the depletion process has influenced the result or removed certain proteins by comparing the results in the two tables.

For the plasma/CSF comparison experiment, a wide range of ratios were observed as described in the results section. The reasons for the different plasma/CSF ratios between plasma-derived proteins remain to be determined, but it has been suggested that factors like protein size, hydrogen bonds and hydrodynamic volume are important for a proteins ability to cross the BBB [29]. Plotting the calculated theoretical MW of the proteins identified in our experiments against the observed plasma/CSF ratio did not show any such correlation (data not shown). The potential ability of the proteins to form multimers/complexes or being associated to larger structures in blood, like cells or platelets, was not taken into account, but is likely to be a main factor in the protein's ability to cross the BBB. This can be exemplified with IgM, apolipoprotein B-100, fibrinogen and haptoglobin which were found to have the highest plasma/CSF ratios in our experiments (Table S6). Based on the %CV values, it was also clear that some proteins had a very varying plasma/CSF ratio between patients (both proteins with high, medium and low plasma/CSF ratio), whereas other proteins displayed less variation in their ratio like complement C3 (15%CV in five individuals). One reason for this could be corresponding variations in plasma of these proteins, but this was not further examined. Of the proteins with plasma/CSF ratio below 0.2, the majority appeared to be CNS-derived proteins like contactin-1 and prostaglandin-H2 D-isomerase, and the contribution of plasma to the CSF concentration of proteins with such low ratios appears to be small.

In theory, the higher the plasma/CSF ratio of a protein, the more influence will the blood contamination have on the CSF concentration. Exceptions would be if there is high intrathecal synthesis of the protein, particularly in combination with low plasma concentrations. As can be observed from Table S7, several of the proteins with high plasma/CSF ratios increase more than five fold from regular CSF to CSF spiked to achieve 2% blood. This includes serum amyloid P-component (11 fold), apolipoprotein C-II (51 fold), apolipoprotein C-III (31 fold) and apolipoprotein M (11 fold). Hence, care should be taken when it comes to interpreting the CSF concentration of proteins with high plasma/CSF ratios, as their CSF concentration appears to be very sensitive to blood contamination.

For the RCG study, the plasma proteins show an increase in concentration towards the lumbar area and steady influx due to diffusion laws are believed to be the cause of this gradient. In contrast to Reiber *et al.* [9], we found none of the selected brain-derived proteins to increase or decrease towards the lumbar area. Our findings are in agreement with the recent publication by Brandner *et al.* [12], where they found that the two CNS-derived proteins S100B and NSE did not decrease from ventricular to lumbar CSF in seven patients with free CSF circulation. Reiber described an increase in concentration in proteins that origins from the leptomeninges that surrounds the spinal cord [9] which was confirmed by Brandner *et al,* using prostaglandin-H2 D-isomerase. Compared to their study, we found the RCG effect on prostaglandin-H2 D-isomerase to be uncertain with a fold change of 1.3 from the first to seventh RCG point. In addition, we verified our iTRAQ results using SID-MRM and included several other brain-derived proteins such as chitinase 3-like protein, secretogranin-3 and S100B and found that none of them decreased down the RCG. Hence, from our results it seems like the concentration of the brain-derived proteins is not affected by the RCG. It should be pointed out that we studied this effect in one patient only, a PSP patient, and examined CSF up to the 44^th^ collected mL which is a limitation of this work. However, our results are supported by the findings by Brander *et al*. [12]. It was also evident from their study as well as ours, that the plasma proteins showed a concentration increase towards the lumbar area. In our study, one could perhaps argue that the gradient is a result of blood entering the sample during LP. However, since the concentration of hemoglobin drops substantially from RGC point 1 to 2, while other high abundant blood proteins such as haptoglobin and fibrinogens are close to linear over the seven RCG points, it strengthens our theory of a gradient. The gradient was also confirmed in two other patients where there was a 30 and 45% change in total protein concentration from the 1^st^ to the 20^th^ mL CSF collected. It therefore seems apparent that the collected volume of CSF affects the protein concentration, and most importantly, that the brain-derived proteins does not appear to be affected. Whether this is true for all conditions and diseases needs to be further tested.

Given the linearity for the plasma proteins in the RCG and that there is a 1.6 fold change (60%) between RCG point 1 (the 1-2^nd^ mL CSF) and 7 (the 44-45^th^ mL CSF), it will be approximately 30% difference in concentration for the plasma proteins if only 1 mL CSF is collected compared to if all 45 milliliters are collected as one sample. In clinical practice between 5 and 20 milliliters of CSF are normally collected, which would give a range of approximately 10-15% variation in the plasma protein concentrations based on the results obtained from our examined patient. This number will, as we have shown, vary from patient to patient, and the effect should be examined in a larger number of patients to get a clear picture of the variation.

When combining the results from all three experimental sections (Table S7) we observed that there is a good correlation between which proteins that were affected by blood spike-in, those that were affected by the RCG and those with relatively high plasma/CSF ratios (> 0.4). There was also a very good correlation between the proteins that were unaffected by both the blood spike-in and the RCG and those with lower plasma/CSF ratios (< 0.2). These observed correlations strengthen the results and conclusions from the individual experiments, and shows that the experiments give complementary but also related information.

## Conclusion

When proteins with high plasma/CSF ratio appear as differentially expressed in CSF-based proteomics experiments, one should strongly consider the chance of this being due to blood contamination. The majority of the CNS-derived proteins in CSF were not affected by blood contamination and the concentration of these proteins could be measured in blood contaminated samples. Most of these proteins also appeared to be unaffected by the RCG, so the volume of CSF collected would not influence the concentration of these proteins in CSF.

The majority of proteins previously suggested as markers for blood contamination, are largely removed from CSF during centrifugation. If the concentration of a detected biomarker candidate is influenced by blood contamination and/or the RCG, then the results should be assessed with this in mind. In addition, the results indicate proteins in CSF which likely arise (fully or partially) from plasma, which is useful information when determining if the concentration of a given CSF biomarker candidate also should be assessed in plasma. In sum, our study gives important information about the influence of factors like blood contamination, RCG and BBB on protein concentration measurements in CSF, particularly important in the field of biomarker discovery, but also for routine clinical measurements. A limitation of our work is the low number of samples included, and future studies including larger number of samples should be conducted to verify our findings.

### Supporting information

The mass spectrometry proteomics data from the blood contamination and RCG discovery studies have been deposited to the ProteomeXchange Consortium (http://proteomecentral.proteomexchange.org) via the PRIDE partner repository [30] with the dataset identifier PXD000401.

## Supporting Information

Document S1 **In-solution digestion protocol.**
(PDF)

Table S1 **Peptides used for quantification in the RCG verification study.** The table contains protein ID, Uniprot accession number, peptide sequence, transition used for quantification, precursor m/z (Q1) and fragment m/z (Q3) of the endogenous and SIS peptides, and collision energy used in the MRM assay. ^a^ indicate that the peptides were of AQUA quality.
(PDF)

Table S2 **Results from the blood contamination study, experiment A.** Proteins from the blood contamination study A (SDS-PAGE label free using Progenesis and analyzed using the database *homo sapiens*, SwissProt) quantified with two or more peptides. CS is the confidence score of the proteins, obtained in the Progenesis LC software. The ratios for 0.5% blood/reference CSF, 2% blood/reference CSF and 0.5% blood/2% blood are calculated. Proteins with ratio >1.5 in either of the two spike-in levels were classified as affected (n = 262). Ratio <1.2 was classified as not affected (n = 403) and ratios in between were uncertain (n = 149). The protein and peptide reports from Progenesis are available in the table.
(XLSX)

Table S3 **Results from the blood contamination study, experiment B.** Comparison of the effect of centrifugation of 0.5% blood contaminated CSF with label free quantification. Ratio >1.5 for 0.5% blood not centrifuged/0.5% blood centrifuged were considered affected by blood contamination, while ratio <1.2 was considered as unaffected. CS is the confidence score from the Progenesis output. The data are identified using the database *homo sapiens*, SwissProt. The protein and peptide reports from Progenesis are available in the table.
(XLSX)

Table S4 **Results from the rostro caudal gradient discovery study.** Results from the iTRAQ discovery experiment applied on the seven RCG points of CSF. Proteins were quantified using Spectrum Mill and identified using homo sapiens, SwissProt. The table lists identification and quantification of 279 proteins with two unique peptides or more with R square value calculated of the seven RCG points, and the fold change (FC) of the 1-2^nd^ and 44-45^th^ mL, corresponding to RCG point 1 and 7. Included in the table is also R squared value of the six RCG points from the 10-11^th^ to the 44-45^th^ mL and the FC between the 10-11^th^ and 44-45^th^ mL (RCG point 2 and 7). The proteins were considered affected or not according to the criteria in Table 1 (also shown in this table under criteria). The protein and peptide reports from Spectrum Mill are available in this table, in addition to a matrix explaining the combination and labeling of experiments 1-3 and a summary of proteins identified with ≥2 peptides.
(XLSX)

Table S5 **Comparison of the R-squared values and fold changes for the peptides quantified with SID-MRM and the iTRAQ experiment for the RCG study.** Proteins are categorized into groups based on the major expected contributing source of origin, Uniprot is the primary reference unless otherwise stated.
(XLSX)

Table S6 **Results from the plasma/CSF study.** Results from the plasma/CSF study (n = 5 patients, P1-P5). The proteins are quantified using dimethyl labeling in Proteome Discoverer (PD), identified using Mascot and the *homo sapiens* database from SwissProt. The average protein ratio, in addition to standard deviation and %CV are included for each protein. The protein and peptide reports from PD are available in the table.
(XLSX)

Table S7 **Summary of Table S2, S3, S4 and S6.** This combined table gives an overview over which proteins that are affected by blood spike-in, centrifugation, RCG and their plasma/CSF ratio, and also how the relation is between the experiments for each protein. The proteins are combined based on accession numbers, except for the iTRAQ discovery study where the leading protein was used.
(XLSX)
