# Supplementary material for: Effects of Blood Contamination and the Rostro-Caudal Gradient on the Human Cerebrospinal Fluid Proteome
Source: PLoS One. 2014 Mar 5;9(3):e90429. doi: 10.1371/journal.pone.0090429 (PMC3943968; doi:10.1371/journal.pone.0090429)
Supplement: Document S1 — In-solution digestion protocol. (PDF) [file pone.0090429.s001.pdf]

In solution protein digestion using trypsin as protease

Urea is a chaotropic agent and disrupts three dimensional structure of proteins and denatures them. ***However, urea + heat + protein = carbamylation;*** *urea in solution is in equilibrium with ammonium cyanate, that may decompose to ammonia and isocyanic acid (HNCO). Isocyanic acid attach the N-terminal of the protein, but also the side chains of lysine and arginine residues rendering a protein unsuitable for many enzymatic digests (HN=C=O + $H_2N\sim \rightarrow H_2N\text{-}CO\text{-}NH\sim$). Urea will always degrade to isocyanic acid, so urea solutions must be made fresh, and it is recommended to add 20mM methylamine (CH_3NH_2)to the urea solution prior to use (urea can also be removed before digestion using reversed phase chromatography)*

Protein solvation/denaturation (applies for 100 µg protein or lower)

Dissolving the protein pellet;

The pellet may be difficult to dissolve. Add **20µl urea solution** *(see right panel)* and pipette gently up and down, sonicate if necessary.

Urea solution; 8M Urea/20mM methylamine:

*Add **480 mg Urea** (art. no. 51458, Sigma-Aldrich), **1.7µl 40 wt% methylamine in H_2O** (art. no. 426466, Sigma-Aldrich) and **630µl dH_2O**.*

Add **20µl trypsin buffer** *(see right panel)*, and incubate at RT in Eppendorf mixer for 5 min (slow agitation).

Trypsin buffer; 50mM Tris/1mM $CaCl_2$:

*Add **0.61g Tris** (art. no. 252859, Sigma-Aldrich) and **15mg $CaCl_2$ x $2H_2O$** (art. no. 21097, Sigma-Aldrich, inhibits chymotrypsin activity) to about 90ml dH_2O. Correct the pH to 7.8-8 with HCl and adjust the volume to 100ml. Store the solution at 4°C.*

Reduction and alkylation

Reduction;

Add **4µl 100 mM DTT** *(see right panel)*, and incubate for 1 hour at room temperature (do NOT use 56˚C as with gel pieces. That will cause carbamylation due to the presence of urea in the sample).

100 mM DTT in MillQ water:

*Add **15.4 mg DTT** (DiThioThreitol, art. no. 171318-02, Amersham Biosciences) to 1ml dH_2O (may be aliquoted as a 1M solution, and kept in freezer).*

Alkylation;

Add **5µl 200 mM IAA** *(see right panel)* for cystein alkylation, and incubate for 1 h at room temperature (dark).

To avoid unwanted protease alkylation, add 0.8µl 100 mM DTT, and incubate 10 min. at room temperature.

200 mM IAA in MilliQ water:

*Add **18.5mg IAA** (Iodoacetamide, art. no. I-6125, Sigma Aldrich) to 0.5ml dH_2O (must be freshly made and kept in the dark).*

Digestion

Sample dilution;

Add 110.2µl Trypsin buffer (the urea concentration is now 1M).

Trypsin;

Add trypsin at a concentration about 50 times lower than the amount of protein in the sample. If the sample contains approx. 100 µg protein, add 2µg of protease *(see right panel)*. Measure pH using an indicator paper (litmus paper or similar), and incubate samples at 37˚C overnight on a shaker

2µg Trypsin Porcine (4µl) *(Promega, art. no. V 5111);*

Dissolve each ampoule (20 µg trypsin porcine) in 40 µl 50 mM acetic acid (resuspension buffer supplied from Promega with the trypsin powder). The trypsin concentration in this stock solution is then 0.5 µg/µl

Acidification

In this final step, add 15 µl 10% FA (formic acid) to quench the digestion activity. We now have approximately 0.5 mg/ml digested protein solution at pH 3. The Urea concentration in this solution (below 1M) allows analysis directly by MALDI or LC-MS. The solution should be desalted/concentrated on reversed phase microcolumns before either MALDI-ToF or nanoflow LC-MS.
