# Supplementary material for: Effects of Blood Contamination and the Rostro-Caudal Gradient on the Human Cerebrospinal Fluid Proteome
Source: PLoS One. 2014 Mar 5;9(3):e90429. doi: 10.1371/journal.pone.0090429 (PMC3943968; doi:10.1371/journal.pone.0090429)
Supplement: Table S1 — Peptides used for quantification in the RCG verification study. The table contains protein ID, Uniprot accession number, peptide sequence, transition used for quantification, precursor m/z (Q1) and fragment m/z (Q3) of the endogenous and SIS peptides, and collision energy used in the MRM assay. a indicate that the peptides were of AQUA quality. (PDF) [file pone.0090429.s002.pdf]

Supplementary Table 1: Name, sequence, transition used for quantification, Q1, Q3 and CE used for MRM analysis

Protein ID	Acc. No	Peptide sequence	Transition used for quantification	Endogenous peptide		SIS peptide		Collision energy
				Q1	Q3	Q1	Q3	
Alpha-1-acid glycoprotein 1	P02763	WFYIASAFR	y7	580.8	827.4	585.8	837.4	21
		TEDTIFLR	y5	497.8	649.4	502.8	659.4	23
Alpha-1-antichymotrypsin	P01011	EIGELYLPK	y5	531.3	633.4	535.3	641.4	19
		ITLLSALVETR	y7	608.4	775.4	613.4	785.4	29
		EQLSLLDR	y5	487.3	603.3	492.3	613.4	23
Alpha-1-antitrypsin	P01009	SVLGQLGITK	y7	508.3	716.4	512.3	724.4	17
Alpha-2-macroglobulin	P01023	AIGYLNTGYQR	y9	628.3	1071.5	633.3	1081.5	35
Amyloid beta A4 protein	P05067	LVFFAEDVGSNK	y10	663.3	1113.5	667.3	1121.5	39
		VESLEQEAANER	y7	687.8	817.4	692.8	827.4	35
Amyloid-like protein 1	P51693	VLLALR	y4	342.7	472.3	347.7	482.3	25
		AALEGFLAALQADPPQAER	y6	984.5	697.4	989.5	707.4	53
Apolipoprotein A-I	P02647	THLAPYSDELR	y7	651.3	879.4	656.3	889.4	32
		DLATVYVDVLK	y6	618.3	736.4	622.4	744.4	21
Apolipoprotein D	P05090	NILTSNNIDVK	y9	615.8	1003.5	619.8	1011.6	24
Apolipoprotein E	P02649	SELEEQLTPVAEETR	y7	865.9	801.4	870.9	811.4	40
Brevican core protein	Q96GW7	GVVFLYR	y4	427.3	598.3	432.3	608.3	13
		YPIVTPSQR	y6	530.8	687.4	535.8	697.4	23
Cell surface glycoprotein MUC18	P43121	GATLALTQVTPQDER[a]	y5	533.9	644.3	537.3	654.3	15
		EVTVPVFYPTEK[a]	y8	704.9	980.5	708.9	988.5	39
		NGYPIPQVIWYK[a]	y9	739.4	1143.7	743.4	1151.7	34
Ceruloplasmin	P00450	GAYPLSIEPIGVR	y8	686.4	870.5	691.4	880.5	37
Chitinase-3-like protein 1	P36222	VTIDSSYDIAK[a]	y9	606.3	1011.5	610.3	1019.5	25
Chromogranin-A	P10645	ELQDLALQGAK	y6	593.3	587.4	597.3	595.4	24
		EDSLEAGLPLQVR	y7	713.9	782.5	718.9	792.5	35
Clusterin	P10909	IDSLLENDR	y5	537.8	646.3	542.8	656.3	23
Complement C3	P01024	ISLPESLK	y5	443.8	573.3	447.8	581.3	17
		NTLIIYLDK	y6	546.8	764.5	550.8	772.5	19
Complement factor H	P08603	SSNLIILEEHLK	y7	698.4	881.5	702.4	889.5	33
Contactin-1	Q12860	FIPLIPIPER	y8	597.9	934.6	602.9	944.6	24
		DGEYVVEVR	y6	533.3	764.4	538.3	774.4	18
Cystatin-C	P01034	ALDFAVGEYNK	y6	613.8	709.4	617.8	717.4	37
		LVGGPMDASVEEEGVR	y8	822.9	904.4	827.9	914.4	45
Disintegrin and metalloproteinase domain-containing protein 22	Q9P0K1	FAISENPLITLR	y9	687.4	1042.6	692.4	1052.6	29
Gelsolin	P06396	EVQGFESATFLGYFK	y7	861.9	875.5	865.9	883.5	48
Haptoglobin	P00738	TEGDGVYTLNNEK	y7	720.3	881.4	724.3	889.5	30
Hemoglobin subunit alpha	P69905	VGAHAGEYGAEALER	y10	765.4	1094.5	770.4	1104.5	44
Hemoglobin subunit beta	P68871	SAVTALWGK	y6	466.8	675.4	470.8	683.4	15
		VNVDEVGGEALGR	y7	657.8	659.3	662.8	669.4	39
Inter-alpha-trypsin inhibitor heavy chain H4	Q14624	GPDVLTATVSGK	y8	572.8	776.5	576.8	784.5	23
Kallikrein-6	Q92876	LSELIQPLPLER	y6	704.4	724.4	709.4	734.4	32
Leucine-rich alpha-2-glycoprotein	P02750	DLLLPQPDLR	y6	590.3	725.4	595.3	735.4	23
N-acetyllactosaminide beta-1,3-N-acetylglucosaminyltransferase	O43505	EPGEFALLR	y4	516.3	472.3	521.3	482.3	33
		TALASGGVLDASGDYR	y8	776.9	896.4	781.9	906.4	44
Neural cell adhesion molecule 1	P13591	GLGEISAASEFK	y7	604.8	739.4	608.8	747.4	27
Neuronal pentraxin receptor	O95502	ELDVLQGR	y4	465.3	473.3	470.3	483.3	31
Neurosecretory protein VGF	O15240	AYQGVAAPFPK	y8	574.8	786.5	578.8	794.5	35
Neuroserpin	Q99574	ALGITEIFIK[a]	y6	552.8	750.4	556.8	758.5	23
		QEVPLATLEPLVK	y10	718.9	1080.7	722.9	1088.7	37
Neurotrimin	Q9P121	VTVNYPPYISEAK	y8	740.9	904.5	744.9	912.5	31
Pigment epithelium-derived factor	P36955	SSFVAPLEK	y5	489.3	557.3	493.3	565.3	19
		DTDTGALLFIGK	y8	625.8	818.5	629.8	826.5	29
ProSAAS	Q9UHG2	GEAAGAVQELAR	y6	586.3	715.4	591.3	725.4	36
		ILAGSADSEGVAAPR	y9	707.4	901.4	712.4	911.4	41
Prostaglandin-H2 D-isomerase	P41222	WFSAGLASNSSWLR	y8	791.4	920.5	796.4	930.5	38
		AQGFTEDTIVFLPQTDK	y7	637.3	848.5	640.0	856.5	22
Protein kinase C-binding protein NELL2	Q99435	AFLFQDTPR	y6	547.8	763.4	552.8	773.4	34
Protein S100-B	P04271	AMVALIDVFHQYSGR	y7	569.6	894.4	573.0	904.4	28
Secretogranin-1	P05060	NYLNYGEEGAPGK[a]	y8	706.3	744.4	710.3	752.4	31
		GEAGAPGEEDIQGPTK	y11	778.4	1170.6	782.4	1178.6	39
Secretogranin-2	P13521	VLEYLNQEK	y7	568.3	923.4	572.3	931.5	21
		IILEALR	y5	414.3	601.4	419.3	611.4	17
Secretogranin-3	Q8WXD2	TEAYLEAIRK	y6	597.3	729.5	601.3	737.5	36
		LNVEDVDSTK	y6	560.3	664.3	564.3	672.3	35
Selenoprotein P	P49908	LPTDSELAPR	y8	549.8	888.4	554.8	898.5	19
Semaphorin-7A	O75326	VYLFDFPEGK	y8	607.8	952.5	611.8	960.5	21
Serotransferrin	P02787	YLGEEYVK	y6	500.8	724.4	504.8	732.4	17
Serum albumin	P02768	LVNEVTEFAK	y6	575.3	694.4	579.3	702.4	27
Serum amyloid P-component	P02743	VFVFPR	y4	382.7	518.3	387.7	528.3	15
Transthyretin	P02766	AADDTWEPFASGK	y8	697.8	921.4	701.8	929.5	25
Vitronectin	P04004	FEDGVLDPDYPR	y10	711.8	1146.5	716.8	1156.6	18
